# The Identity of Bovine and Human Tuberculosis

**Published:** 1882-07

**Authors:** 


THE IDENTITY OF BOVINE AND HUMAN TUBERCULOLIS has been tested again with inconclusive results by Professor Pütz, of the Veterinary College of Halle. He experimented upon five animals, injecting into their tissues tuberculous fluids obtained from men. On February 6th, he injected half an ounce of tuberculous matter from a human lung beneath the skin of a bull-calf. A week later he made a similar injection directly into the lung tissue. The animal was killed six months later and its tissues found to be perfectly healthy.

A horse had injected under the skin of the chest, ʒ½i of pus from a tuberculous abscess. Three weeks later ʒss. more was injected in two places. A pig was injected once with the same matter. Two weeks after the last injection the animals were killed and found to be slightly tuberculous.

A ten months old calf was inoculated with no result. A colt had tuberculous matter injected directly into his lungs. The result was the rapid development of an acute tuberculosis.

The experiments throw some doubt over the exact identity of human and bovine tuberculosis, or at least over the absolute infectiousness of the former.

